# Feasibility and Efficacy of Using Hem-o-lok Polymeric Clips in Appendicular Stump Closure in Laparoscopic Appendectomy

**DOI:** 10.7759/cureus.2871

**Published:** 2018-06-23

**Authors:** Gigi Varghese

**Affiliations:** 1 Surgery, Christian Medical College, Vellore, IND

**Keywords:** laparoscopic appendectomy, surgery

## Abstract

Laparoscopic appendectomy is becoming the gold standard for the management of acute appendicitis. Appendicular stump closure is a critical step in this procedure, and several methods have been used to secure the appendicular stump during laparoscopic appendectomy. We highlight the feasibility and efficacy of ‘Hem-o-lok’ polymeric clips in securing the base of the appendix. We selected 20 consecutive patients who underwent laparoscopic appendectomy using a single or double ‘Hem-o-lok’ clip to secure the base of the appendix for acute appendicitis from October 2011 to August 2013. Thirteen study participants were men, and seven were women. There were no instances of clip slippage, obstruction due to adhesion to the clip or postoperative collections. Laparoscopic appendectomy using polymeric ‘Hem-o-lok’ clips to secure the base of the appendix is a feasible and efficacious option.

## Introduction

Laparoscopic appendectomy is gradually evolving as the ‘gold standard’ in the treatment of acute appendicitis, especially in the obese, elderly, and in cases where the diagnosis is uncertain [1,2]. There are clear benefits with the laparoscopic approach in terms of decreased pain and faster recovery than the open approach [3]. However, disadvantages like a higher incidence of intra-abdominal collection, longer operating time, and higher costs have been documented. These may be dependent on the technique of appendicular stump closure. Therefore, the technique for appendiceal stump closure is crucial in laparoscopic appendectomy [4-6].

Laparoscopic appendectomy was first described by Semm in 1983 and is currently preferred approach for an appendectomy for many surgeons. Numerous methods of stump closure have been used, like intracorporeal suturing of the stump [7], endoloop application, endostapler application [8], LigaSure [9], customized titanium clip application, and, more recently, using nonabsorbable plastic clips [7,10]. Many of these techniques are either time consuming or expensive, or not available universally.

We aimed to assess the feasibility and efficacy of closure of the appendicular stump during laparoscopic appendectomy using the Weck® Hem-o-lok® Polymer Locking Ligation System (Weck, Research Triangle Park, NC; i.e., Hem-o-lok clips), which are universally available and easy to apply.

## Materials and methods

Twenty consecutive patients who underwent laparoscopic appendectomy as an elective or emergency procedure from October 2011 and August 2013 were included. Appropriate preoperative written informed consent was obtained. We retrospectively collected and analyzed data from computerized hospital records. Patient demographic data are presented in Table 1.

**Table 1:** Laparoscopic appendectomy demographics.

Demographic	N
Sex	
Men	13
Women	7
Urgency of Operation	
Emergency	19
Elective	1
Histopathology	
Acute Appendicitis	16
Perforated Appendicitis	2
Necrotizing Appendicitis	1
Lymphoid Hyperplasia	1
Mean Duration of Postoperative Hospital Stay (days; range)	2.05; 1-5

Operative procedure

The patient was positioned supine. After placement of laparoscopic ports, a slight head down and right-sided up position was employed. The surgeon and assistant stood on the patient’s left, and the monitor was positioned towards the patient’s right hip. A Foley catheter was inserted preoperatively for continuous bladder drainage and later removed immediately after the operation or on the following day.

**Figure 1:**
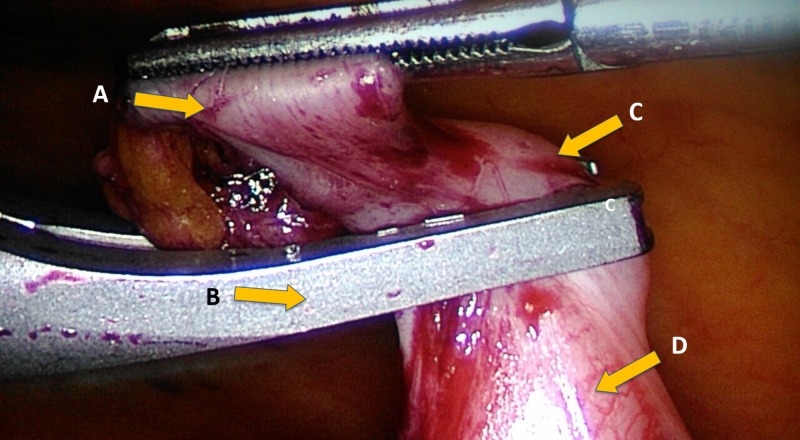
Appendicular base prior to deploying Hem-o-lok clips. (A) Appendix; (B) Hem-o-lok applicator; (C) Appendicular base; (D) Caecum.

**Figure 2:**
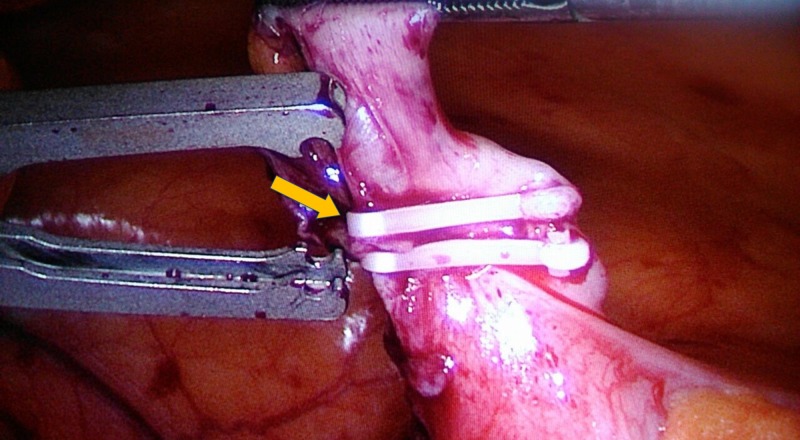
Appendicular base after application of two Hem-o-lok polymeric clips. Appendicular base after deploying two Hem-o-lok polymeric clips (arrow) at the appendicular base prior to dividing the appendix.

After induction of general anesthesia, pneumoperitoneum was established using a 10-mm or 11-mm subumbilical port inserted by the open method. A 10-mm, 30-degree laparoscope was used to explore the peritoneal cavity via the umbilical port. Thereafter, under direct vision, a 5-mm suprapubic port and a 10-mm or 11-mm port were inserted in the left lower quadrant. The laparoscope would then be shifted to the left lower quadrant port, and the other two ports used as working ports, the umbilical port being the main working port. The appendix was then held and retracted using a grasper, and the mesoappendix dissected using hook and cautery. Occasionally a Weck Hem-o-lok Polymer Locking Ligation System was used to clip the appendicular artery on the patient side and divided using monopolar hook and cautery. Once the appendix was free of its mesoappendix, the appendicular base was clearly visualized. The appendix was then divided using laparoscopic scissors between two Hem-o-lok clips. In some cases, two Hem-o-lok clips were used on the patient side. The specimen was then removed via the umbilical port. The technique of application of Hem-o-lok clips at the base of the appendix is shown in Figures 1, 2.

Patients were discharged by the second or the third postoperative day after assessment by the surgical team involved. Skin clips or sutures were removed during the out-patient follow-up evaluation.

## Results

Of the 20 patients included, 13 were men and seven were women. The mean age was 27 years (range, 16-45 years). Nineteen patients had emergency appendectomy for suspected acute appendicitis, and one patient had elective interval appendectomy. Histopathological examination revealed a diagnosis of acute appendicitis in 16 patients, perforated appendicitis in two patients, necrotizing appendicitis in one patient, and acute appendicitis with lymphoid hyperplasia in one patient. There were no intraoperative complications.

Mean postoperative hospital stay was 2.05 days (range, 1-5 days). Fourteen patients were discharged within two days, and the longest postoperative hospital stay was five days due to the need for re-laparoscopy. One patient had persistent abdominal pain, for which no cause was found. One patient was readmitted for conservative management of a right iliac fossa mass on follow-up in the out-patient clinic seven days postoperatively. The mass subsided on conservative management, and the patient was discharged on the sixth day following readmission. Three patients were lost to follow-up. Mean follow-up was 11.4 days (range, 7-40 days). There were no instances of clip slippage, obstruction due to adhesion to the clip or postoperative collections.

## Discussion

Laparoscopic appendectomy is expected to gain acceptance among surgeons as the ‘gold standard’ operation for acute appendicitis, similar to the evolution of laparoscopic cholecystectomy [11]. Several studies have shown statistically significant benefits like lower instances of wound infection, less postoperative pain, and shorter postoperative hospital stays when compared to the open appendectomy approach. Despite these advantages, there is an increased incidence of postoperative intra-abdominal collections after laparoscopic appendectomy [12]. Whether the higher incidence of intra-abdominal collections is related to the technique of stump management is debatable. Various methods of appendicular stump closure have been employed such as intracorporeal suturing, endoloop application, titanium clip application, ligature shears, metal ring application [13], endostapler application, and plastic clip application.

Several studies have compared two or more of these methods in appendicular stump management [7,8, 14-17]. Most of these techniques are time-consuming, expensive or not widely available because they are custom made [4]. Endostaplers and commercially available endoloops are expensive and are of limited use in countries where the final cost is a major concern [14,15]. Surgeon-made endoloop ligatures using catgut or polydioxanone suture materials are cumbersome and require prolonged application time, resulting in prolonged operation time.

Hem-o-lok clips have been widely used in various laparoscopic procedures. The safety of Hem-o-lok clip use has been demonstrated for ligating the cystic duct, ureter, and vessels up to 16 mm in diameter [18]. The sizes of Hem-o-lok clips available are M (blue), ML (green), L (purple), to XL (brown) capable of ligating tissue bundle sizes up to 16 mm. The laparoscopic applicator for Hem-o-lok clips can be used through a 10-mm port and is easy to deploy (Poster: Aminian A, Karimian F, Mirsharifi R. Application of Hem-o-lok Clip in Basic Laparoscopic Procedures: A Single Center Experience on 856 Cases and Review of Data From FDA. 12th World Congress of Endoscopic Surgery. April 14-17, 2010).

The use of Hem-o-lok clips in ligating the base of the appendix involves no learning curve and is easily replicable (Poster: Aminian A, 2010). We found no instances of clip slippage, obstruction due to adhesion to the clip or postoperative collections. A further randomized controlled trial is necessary to compare the various methods of appendicular stump management.

## Conclusions

Hem-o-lok clips are a feasible and efficacious option to secure the base of the appendix for laparoscopic appendectomy.
